# Quantitative analysis of the ACL and PCL using T1rho and T2 relaxation time mapping: an exploratory, cross-sectional comparison between OA and healthy control knees

**DOI:** 10.1186/s12891-021-04755-y

**Published:** 2021-10-30

**Authors:** Chanuka D. S. Ranmuthu, James W. MacKay, Victoria A. Crowe, Joshua D. Kaggie, Dimitri A. Kessler, Stephen M. McDonnell

**Affiliations:** 1 School of Clinical Medicine, Addenbrooke’s Hospital, University of Cambridge, Cambridge, CB2 0SP UK; 2 London, UK; 3 Department of Radiology, Addenbrooke’s Hospital, University of Cambridge, Cambridge, CB2 0QQ UK; 4 Norwich Medical School, University of East Anglia, Norwich Research Park, Norwich, NR4 7UY UK; 5 Cambridge University Hospitals NHS Foundation Trust, Addenbrooke’s Hospital, Cambridge, CB2 0QQ UK; 6 Division of Trauma & Orthopaedic Surgery, Addenbrooke’s Hospital, University of Cambridge, Cambridge, CB2 0QQ UK

**Keywords:** T1rho mapping, T2 mapping, Anterior cruciate ligament, Posterior cruciate ligament, Osteoarthritis

## Abstract

**Background:**

Quantitative magnetic resonance imaging (MRI) methods such as T1rho and T2 mapping are sensitive to changes in tissue composition, however their use in cruciate ligament assessment has been limited to studies of asymptomatic populations or patients with posterior cruciate ligament tears only. The aim of this preliminary study was to compare T1rho and T2 relaxation times of the anterior cruciate ligament (ACL) and posterior cruciate ligament (PCL) between subjects with mild-to-moderate knee osteoarthritis (OA) and healthy controls.

**Methods:**

A single knee of 15 patients with mild-to-moderate knee OA (Kellgren-Lawrence grades 2–3) and of 6 age-matched controls was imaged using a 3.0 T MRI. Three-dimensional (3D) fat-saturated spoiled gradient recalled-echo images were acquired for morphological assessment and T1ρ- and T2-prepared pseudo-steady-state 3D fast spin echo images for compositional assessment of the cruciate ligaments. Manual segmentation of whole ACL and PCL, as well as proximal / middle / distal thirds of both ligaments was carried out by two readers using ITK-SNAP and mean relaxation times were recorded. Variation between thirds of the ligament were assessed using repeated measures ANOVAs and differences in these variations between groups using a Kruskal-Wallis test. Inter- and intra-rater reliability were assessed using intraclass correlation coefficients (ICCs).

**Results:**

In OA knees, both T1rho and T2 values were significantly higher in the distal ACL when compared to the rest of the ligament with the greatest differences in T1rho (e.g. distal mean = 54.5 ms, proximal = 47.0 ms, *p <* 0.001). The variation of T2 values within the PCL was lower in OA knees (OA: distal vs middle vs proximal mean = 28.5 ms vs 29.1 ms vs 28.7 ms, *p =* 0.748; Control: distal vs middle vs proximal mean = 26.4 ms vs 32.7 ms vs 33.3 ms, *p =* 0.009). ICCs were excellent for the majority of variables.

**Conclusion:**

T1rho and T2 mapping of the cruciate ligaments is feasible and reliable. Changes within ligaments associated with OA may not be homogeneous. This study is an important step forward in developing a non-invasive, radiological biomarker to assess the ligaments in diseased human populations in-vivo.

**Supplementary Information:**

The online version contains supplementary material available at 10.1186/s12891-021-04755-y.

## Background

The cruciate ligaments play a critical role in stabilising the knee joint. The anterior cruciate ligament (ACL) has been studied extensively, due to isolated ACL tears being a frequent injury in orthopaedics with an annual incidence of 68.6 per 100,000 persons [1]. Posterior cruciate ligament (PCL) injury is rarer with an estimated annual incidence of 2 per 100,000 persons [2]. Studies have also found that degeneration of the cruciate ligaments (ACL and PCL) may contribute to the onset and progression of other musculoskeletal diseases such as osteoarthritis (OA) [3, 4].

Conventional magnetic resonance imaging (MRI) can depict tears of the cruciate ligaments and the advanced stages of (mucoid) degeneration. However, the sensitivity of conventional MRI is limited in detecting early ligamentous changes and mainly provides qualitative information. Quantitative MRI measurements such as T1rho and T2 have predominantly been used as a way of quantifying proteoglycan, collagen and water content in articular cartilage [5–7]. T1rho and T2 relaxometry have been used extensively to probe the composition of collagen rich tissues such as cartilage, ligament and meniscus [8]. These methods may provide a way of quantitatively assessing changes in the cruciate ligaments before gross morphological changes have occurred. There has been a new emphasis on biological augmentation of the ACL, using growth factors [9], stem cells [10] and bio-scaffolds [11] to regenerate the ACL. Quantitative MRI may also be helpful in such studies looking at understanding and monitoring the biological changes that occur in ligaments post augmentation or repair. Newer techniques have used 3D cartilage surface mapping to analyse these cartilage changes [12]. Furthermore, Prasad et al. used a longitudinal approach and found that T1rho and T2 measurements may predict progression of knee OA, in particular degenerative cartilage abnormalities [13]. However, their in-vivo application in ligaments has been limited to evaluating T2 of the PCL in an asymptomatic population, in patients with PCL tears as well as in ACL’s of asymptomatic populations and individuals post ACL reconstruction [14–17].

To our knowledge, these techniques have not been applied to subjects with OA. The purpose of this study was to evaluate the intra-ligamentous differences in T1rho and T2 values of the ACL and PCL between patients with OA and age-matched healthy controls.

## Methods

### Subjects cohort

The subject cohort used in this cross-sectional study has previously been described in a study assessing longitudinal changes in cartilage morphology and composition [12]. Between April 2017 and June 2018, 15 patients with knee OA were recruited from specialist orthopaedic knee clinics at a University teaching hospital. Six age-matched healthy controls were recruited via paper and electronic advertisement materials and from a register of healthy individuals who had agreed to be contacted about research studies. Key inclusion criteria for OA subjects were: (i) age 40–60 years, (ii) body mass index (BMI) of ≤35 kg/m^2^, (iii) clinical diagnosis of knee OA per American College of Rheumatology criteria [18], and (iv) mild-moderate radiographic OA defined as Kellgren-Lawrence grade 2 or 3 on a postero-anterior fixed flexion knee radiograph taken using a positioning device (SynaFlexer; BioClinica, Newtown, PA) with medial compartment predominant disease [19]. Key exclusion criteria were any history of previous lower limb fracture, previous knee surgery (including arthroscopy) or history of inflammatory arthritis. For control subjects, key inclusion criteria were: (i) age 40–60 years, (ii) no current or significant previous symptoms of knee pain or stiffness and (iii) BMI ≤ 35 kg/m^2^. A MRI Osteoarthritis Knee Score (MOAKS) was additionally conducted on the control subjects to assess any signs of degeneration [20]. At each study visit subjects completed the knee injury and osteoarthritis outcome score (KOOS) to assess symptoms and had their BMI recorded [21].

### Image acquisition

Subjects were imaged using a 3.0 T MRI system (GE 750, GE Healthcare, Waukesha, WI, USA) with an 8 channel transmit/receive dedicated knee coil (InVivo, Gainesville, FL, USA). Subjects were positioned foot first in the supine position with immobilisation of the knee to reduce potential motion artefact. A standard clinical MRI examination protocol including a sagittal 3D fat-saturated spoiled gradient recalled-echo (3D-FS SPGR) sequence and additional sagittal T1rho and T2 mapping sequences (pseudo steady state 3D fast spin echo sequences with T1ρ and T2 magnetisation preparation) was used. A summary of key sequence parameters is provided in Table 1.

**Table 1:** Sequence parameters

Sequence	TR (ms)	TE (ms)	TSL (ms)	Matrix	FOV (mm)	In-plane spatial resolution (mm)	Slice thickness (mm)	Flip angle (^o^)	No. of averages	Sequence duration (mins)
3D-FS SPGR	25.6	6.7	N/A	512 × 380	160 × 120	0.31 × 0.31	1	20	0.5	7
3D FSE T1rho	1260	35	1/10/20/35	320 × 256	150 × 120	0.47 × 0.47	3	90	0.5	5
3D FSE T2	1285	6.5/13.4/27.0/40.7	N/A	320 × 256	150 × 120	0.47 × 0.47	3	90	0.5	5

*NOTE: TR* Repetition time, *TE* Echo time, *TSL* Spin-lock pulse duration, *FOV* Field of view, *SPGR* Spoiled gradient recalled-echo, *FS* fat-suppressed, *FSE* Fast spin echo. No. of averages = 0.5 = a half-fourier acquisition using phase-conjugate symmetry for acceleration purposes. Sequence times are rounded to nearest 30 s

### Image analysis

**Fig. 1:**
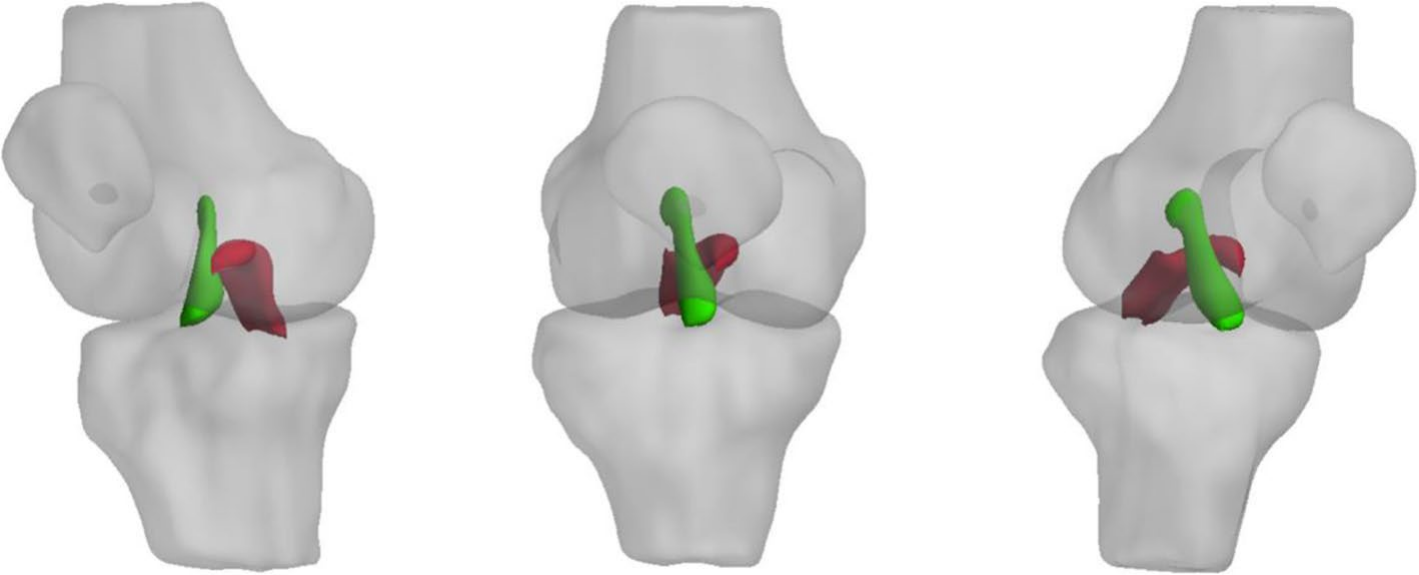
Representative 3D renderings of ACL(green) and PCL (red) segmentations superimposed on 3D models of the knee joint and viewed from (from left to right) anteromedially, anteriorly and anterolaterally

The ACL and PCL were segmented manually on the high spatial resolution anatomical 3D-FS SPGR images using ITK-SNAP v 3.6.0 [22]. Segmentation was performed by a single reader (CDSR) and supervised by a musculoskeletal radiologist with 8 years’ experience (JWM). Segmentations were repeated by the same reader at a second timepoint (at least 2 weeks following the initial segmentation) to assess intra-rater reproducibility, and also by an independent second reader, a radiologist with 3 years’ experience (VC), to assess inter-rater reproducibility. Segmentation was done on 5–10 sagittal images corresponding to 2–3 images on T1rho/T2 maps. An example of a 3D- rendering made from a segmentation in ITK-SNAP is shown in Fig. 1.

Images for T1rho and T2 mapping (individual spin-lock time/echo times) were rigidly registered to the 3D-FS SPGR images using Elastix registration software [23]. Parameter maps were constructed from the registered images by fitting an exponential decay curve to the observed signal on a voxel-by-voxel basis using non-linear least squares curve fitting with a routine developed in MATLAB (R2017a, Mathworks) in the regions masked by the ACL/PCL segmentations. The fitting routine excluded pixels from the final map if the signal at any spin lock/echo time was less than the estimated noise floor or if the curve fit was poor (operationally defined as R^2^ < 0.8). The mean and standard deviations T1rho and T2 relaxation times within the segmented masks for the ACL and PCL were then recorded. The cruciate ligaments were also divided into thirds (proximal, middle and distal) based on their maximal length in the craniocaudal direction using an in-house MATLAB routine utilising the manual segmentation to define the proximal and distal extent of the ligaments. Mean T1rho and T2 relaxation times were also recorded separately for each third.

### Additional exploratory analyses

We conducted additional exploratory analyses between OA individuals who had a bone marrow lesion associated with the ACL/PCL tibial insertion and those without ganglion cysts at the footplates/footprints. We also conducted comparisons between OA individuals with KL Grade 3 and those with KL Grade 2. These can be found in the Additional file 1.

### Statistical analysis

To compare structural variation across the thirds of the ligament for each group, repeated measures ANOVAs were performed. Any violations in assumptions were checked before comparison. If there was a violation of sphericity or normality, a Greenhouse-Geisser correction was applied or a Friedman test was used, respectively. To investigate whether this structural variation differs between groups (OA vs CON), percentage difference values were obtained between each of the thirds of the ligament. These percentage difference values were then compared across groups using a Kruskal-Wallis test. Intra-rater and inter-rater reproducibility of the extracted mean T1rho and T2 relaxation times were assessed using intraclass correlation coefficients (ICCs). For inter-rater agreement, a two-way random effects, single measures model was used. For intra-rater agreement, a two-way mixed effects, single measures model was used. Both models looked for absolute agreement. Any ICC below 0.4 was deemed to represent poor agreement; one between 0.4 and 0.75 represented fair to good agreement; an ICC above 0.75 was interpreted as “excellent agreement.” Data from the first reader’s segmentation was used for all other analyses. SPSS (IBM Corp, Version 25) was used for statistical analysis. We did not correct for multiplicity of testing in this exploratory study.

## Results

### Subject characteristics

We investigated 15 symptomatic subjects in our knee OA sample and 6 control subjects (CON). Subject demographics are shown in Table 2. Four of the individuals in the OA group had a K-L grade of 3 and eleven had a K-L grade of 2. The results of the structural, semi-quantitative assessment of the healthy controls using MOAKS scoring are presented in Table 3.

**Table 2:** Demographics of the sample

Group	Control (*N* = 6; 4 F, 2 M)		OA (*N* = 15; 6 F, 9 M)	
	Mean	SD	Mean	SD
Age (years)	55.0	3.5	51.1	5.0
BMI (kg/m^2^)	29.7	3.0	29.2	3.9
KOOS-5	91.8	15.8	50.9	17.7

*NOTE*: *N* number in sample, *F* Female, *M* Male, *SD* Standard deviation, *BMI* Body Mass Index, *KOOS-5* Knee Injury and Osteoarthritis Outcome Score, average across 5 subscales

**Table 3:** MRI Osteoarthritis Knee Score (MOAKS) for Healthy Controls

MOAKS domain	Number of healthy controls (n = 6) with feature
**Bone marrow lesion**	
- Any	1
- ≥ grade 2	0
**Cartilage defect**	
- Any	2
- ≥ grade 2 (size)	1
- Any full-thickness	1
**Osteophyte**	
- Any	2
- ≥ grade 2	0
**Meniscal damage**	
- Any medial tear	1
- Any lateral tear	1
**Meniscal extrusion**	
- Any medial	4
- ≥ grade 2 medial	4
- Any lateral	1
- ≥ grade 2 lateral	0
**Effusion-synovitis**	
- Any	1
- ≥ grade 2	0
**Hoffa-synovitis**	
- Any	1
- ≥ grade 2	0

### Variation across ligaments

**Fig. 2:**
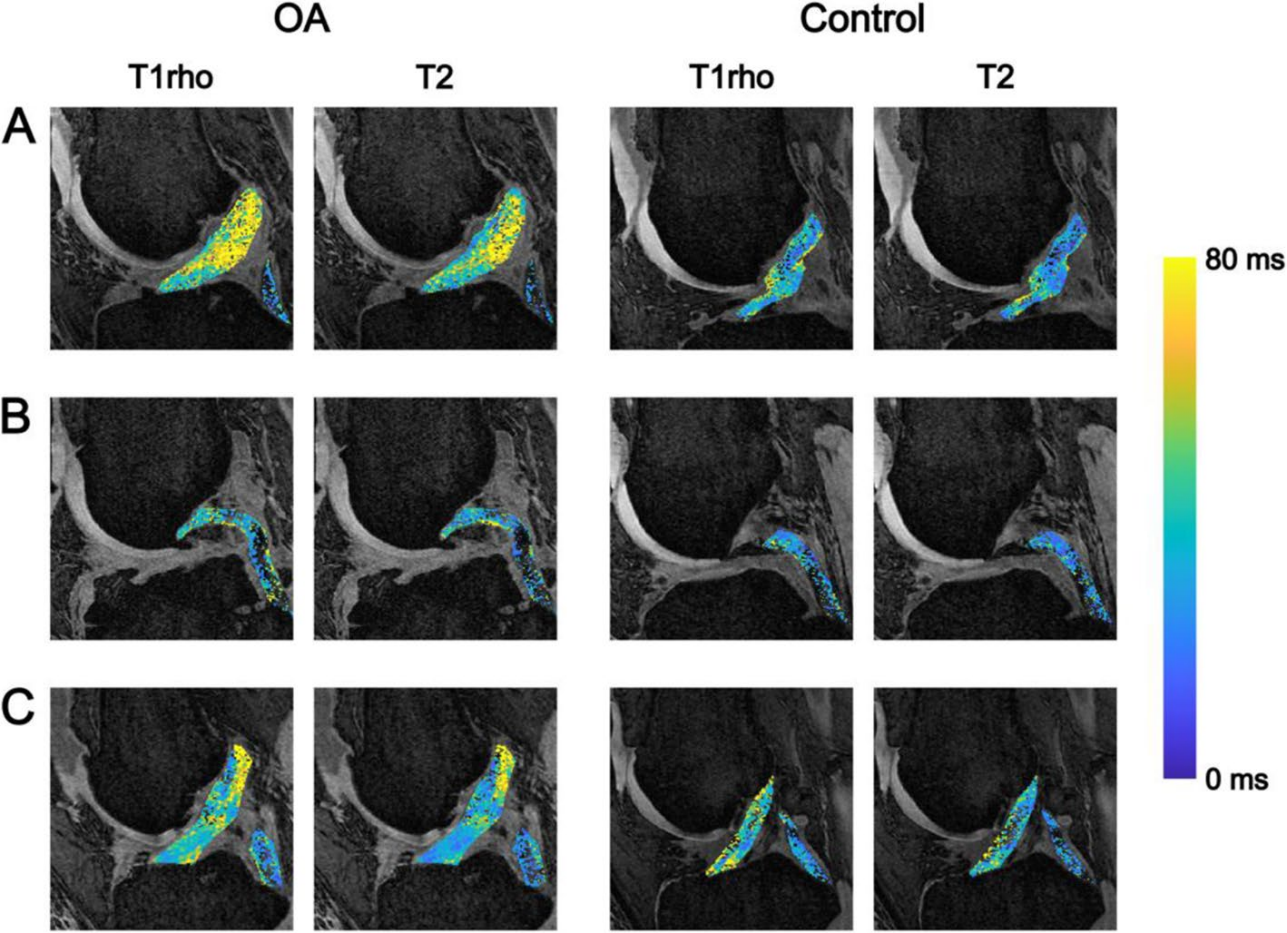
**A** Representative sagittal 3D spoiled gradient echo MRI images of the ACL of an OA (left) and healthy (right) subject with overlaid T1rho colormaps. Note higher and more heterogeneous values of T1rho in the OA subject. **B** As above but with T1rho colormaps of the PCL. Note higher T1rho values in proximal part of the PCL in the OA subject. **C** Variation in T1rho within the cruciate ligaments of an OA (left) and healthy (right) subject. Note increased variation/heterogeneity of T1rho values throughout the ACL of the OA subject

**Fig. 3:**
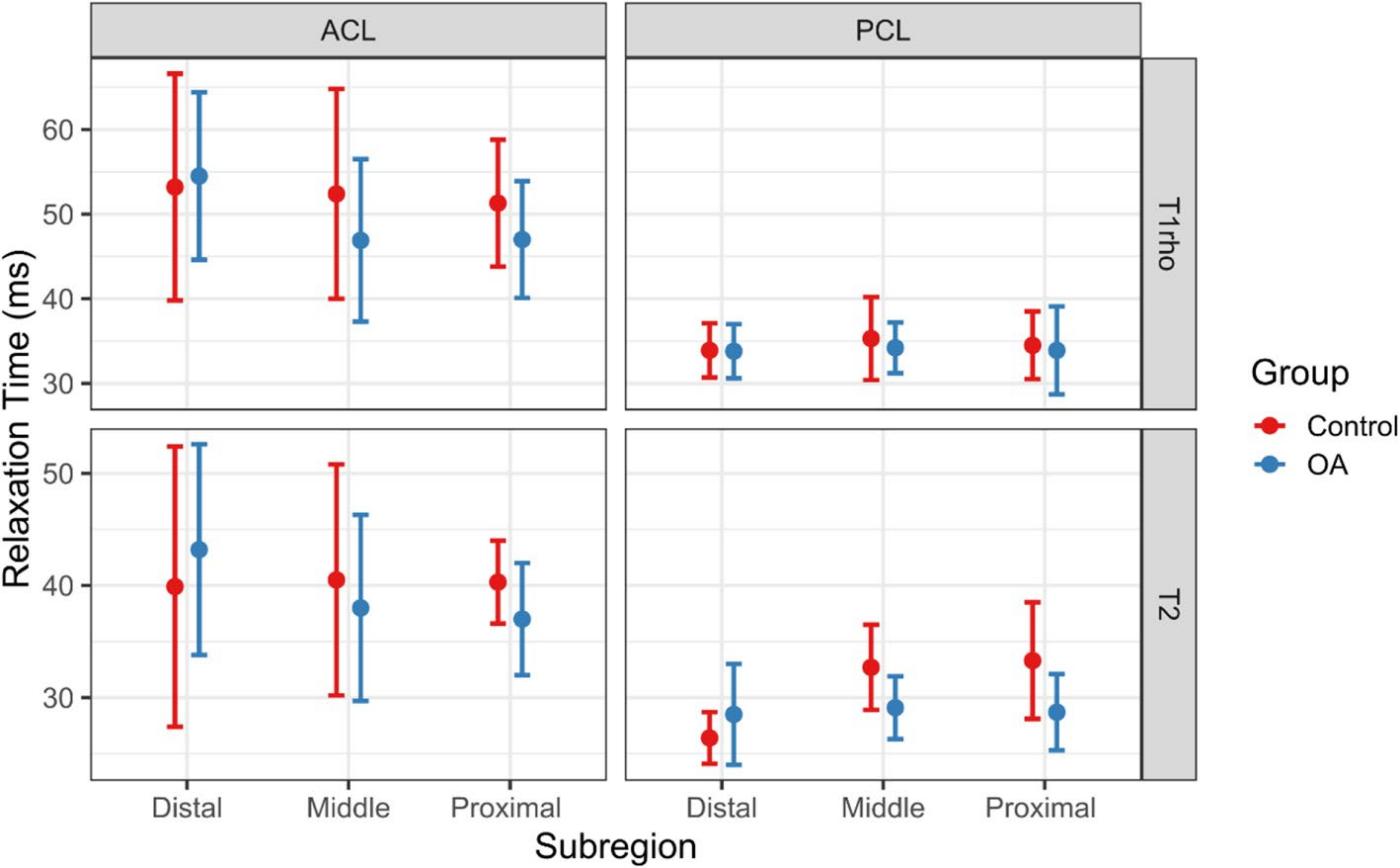
Means and standard deviations of T1rho and T2 relaxation time measurements by ACL and PCL subregion for OA and healthy control subjects. Note: ACL = anterior cruciate ligament; PCL = Posterior cruciate ligament

Examples of T1rho colour maps for the ACL and PCL in healthy and OA individuals are shown in Fig. 2. Means and standard deviations for each parameter (T1rho and T2), subregion (distal, middle and proximal thirds of the ligament) and group (OA and control) are presented in Table 4. The data is also displayed graphically in Fig. 3. Two variables for the OA group displayed significant variation across the ligament: mean T1rho times of the ACL (distal = 54.5 ± 9.9 ms, middle = 46.9 ± 9.6 ms, proximal = 47.0 ± 6.9 ms; *p <* 0.001) and mean T2 times of the ACL (distal = 43.2 ± 9.4 ms, middle = 38.0 ± 8.3 ms, proximal = 37.0 ± 5.0 ms; *p =* 0.017). Pairwise comparisons revealed for the former that the distal third of the ligament varied significantly from both the middle and the proximal thirds (*p =* 0.007 and 0.002 respectively). Pairwise comparisons for the latter revealed that the distal third of the ligament varied significantly from the proximal third (*p =* 0.007). For the control group, the mean T2 relaxation times for the PCL varied significantly across the ligament (distal = 26.4 ± 2.3 ms, middle = 32.7 ± 3.8 ms, proximal = 33.3 ± 5.2 ms; *p =* 0.009). Pairwise comparisons revealed again that the distal third of the ligament varied significantly from both the middle and the proximal third (both *p =* 0.031).

**Table 4:** Summary of T1rho and T2 variables by ACL and PCL subregion for OA and controls

			Distal		Middle		Proximal		Variation across ligament	
Group	Ligament	Parameter	Mean (SD)	Sig.	Mean (SD)	Sig.	Mean (SD)	Sig.	*p-values*	Effect size
OA	ACL	T1rho	54.5 (9.9)	M, P	46.9 (9.6)	D	47.0 (6.9)	D	**< 0.001**	0.430
		T2	43.2 (9.4)	P	38.0 (8.3)		37.0 (5.0)	D	0.017*	0.271***
	PCL	T1rho	33.8 (3.2)		34.2 (3.0)		33.9 (5.2)		0.881**	0.005
		T2	28.5 (4.5)		29.1 (2.8)		28.7 (3.4)		0.748	0.021
Controls	ACL	T1rho	53.2 (13.4)		52.4 (12.4)		51.3 (7.5)		0.855	0.031
		T2	39.9 (12.5)		40.5 (10.3)		40.3 (3.7)		0.987	0.003
	PCL	T1rho	33.9 (3.2)		35.3 (4.9)		34.5 (4.0)		0.790	0.046
		T2	26.4 (2.3)	M, P	32.7 (3.8)	D	33.3 (5.2)	D	0.009*	0.778***

NOTE: Means and standard deviations (SD) are in ms. Within the (Sig.) column: M = different from middle; P = different from proximal; D = different from distal (all *p* < 0.05); * = A Friedman test was used here; ** = *p*-value after Greenhouse-Geiser correction; all effect sizes are partial-eta squared values unless indicated by a *** which represents a Kendall’s co-efficient of concordance value; *p*-values < 0.05 are shown in **bold**. ACL = Anterior cruciate ligament; PCL = Posterior cruciate ligament

### Comparing the variation across the ligament between groups (OA vs control)

Table 5 shows the mean difference between T1rho ACL and T2 PCL of each group for each variable. A significant difference between groups was found for the variation in mean T2 values between the distal and middle thirds of the PCL as well as the variation in mean T2 values between the distal and proximal thirds of the PCL (*p =* 0.006 and 0.008 respectively).

**Table 5:** Summary of differences in variation of T1rho and T2 variables across the ligaments between groups (OA vs Control) using a Kruskal-Wallis test

Ligament	Parameter	Difference between Regions	OA mean rank	Control mean rank	Kruskal-Wallis H (df = 1)	Sig. (two-tailed)
ACL	T1rho	D and M	12.5	7.3	2.93	0.087
		D and P	12.7	6.8	3.79	0.052
	T2	D and M	12.3	7.7	2.42	0.119
		D and P	12.3	7.7	2.42	0.119
PCL	T1rho	D and M	11.0	11.0	0.00	1.000
		D and P	11.3	10.3	0.10	0.755
	T2	D and M	13.3	5.2	7.42	**0.006**
		D and P	13.3	5.3	7.01	**0.008**

NOTE: *p*-values < 0.05 are shown in **bold**

D and M = Difference between the T1rho and T2 value for the distal part of the ligament and the Middle part; D and P = Difference between the T1rho and T2 value for the distal part of the ligament and the Proximal part; OA = OA group; CON = Control group; ACL = Anterior cruciate ligament; PCL = Posterior cruciate ligament

### Inter- and intra-rater reliability

Intra-rater and inter-rater reliability was assessed for all T1rho and T2 variables for the three sub-regions and for the ligament as a whole using the intraclass correlation co-efficient (ICC) within subjects and between subjects respectively [24]. The values are shown in Table 6. The majority of variables were deemed to have “excellent agreement.”

**Table 6:** Results of intra-rater and inter-rater reliability assessment

**Intra-rater reliability**	**T1rho**		**T2**	
	**ACL (95% CI)**	**PCL (95% CI)**	**ACL (95% CI)**	**PCL (95% CI)**
Overall	0.94 (0.86, 0.98)	0.78 (0.53, 0.91)	0.92 (0.81, 0.97)	0.84 (0.65, 0.93)
Distal	0.58 (0.21, 0.81)	0.68 (0.36, 0.86)	0.61 (0.24, 0.82)	0.81 (0.58, 0.92)
Middle	0.83 (0.62, 0.93)	0.79 (0.54, 0.91)	0.80 (0.57, 0.92)	0.77 (0.51, 0.9)
Proximal	0.89 (0.74, 0.96)	0.75 (0.47, 0.89)	0.92 (0.77. 0.97)	0.68 (0.35, 0.86)
**Inter-rater reliability**	**T1rho**		**T2**	
	**ACL**	**PCL**	**ACL**	**PCL**
Overall	0.91 (0.66, 0.97)	0.76 (0.46, 0.91)	0.84 (0.61, 0.94)	0.84 (0.62, 0.94)
Distal	0.33 (−0.92, 0.68)	0.2 (−0.35, 0.63)	0.5 (0.04, 0.78)	0.78 (0.48, 0.91)
Middle	0.83 (0.56, 0.94)	0.75 (0.39, 0.91)	0.83 (0.59, 0.94)	0.86 (0.66, 0.95)
Proximal	0.87 (0.68, 0.95)	0.79 (0.5, 0.92)	0.73 (0.39, 0.89)	0.31 (−0.18, 0.68)

NOTE: Overall = The inter/intra-rater reliability across the whole of the ligament. *ACL* Anterior cruciate ligament, *CI* Confidence interval, *PCL* Posterior cruciate ligament

## Discussion

### Validation of the quantitative MRI technique for cruciate ligament compositional analysis

This is the first study to our knowledge that compares T1rho and T2 relaxation times in the cruciate ligaments between OA and healthy knees. This study has demonstrated the feasibility of segmentation and T1rho and T2 mapping of the cruciate ligaments. Our results indicate that quantitative MRI assessment of the cruciate ligaments is feasible and reliable and may be of clinical utility in diseases such as osteoarthritis. Intra-rater and inter-rater reproducibility was in the “excellent” range for the majority of measurements. However, there were three variables in the “poor range” (These were the inter-rater reliabilities for the T1rho variables of the distal ACL and PCL and the proximal PCL T2 variable). This may be due to inter-observer variation in defining the ligament/bone boundary at proximal/distal attachment sites. This is particularly difficult for the distal ACL for example, as it has a relatively wide fan-shaped attachment which blends in with adjacent lateral meniscal tissue.

### Sub-regional differences in the distribution of T1rho and T2 values between controls and OA groups

A key finding of this study was that there were significant differences in the distribution of T1rho and T2 values of the cruciate ligaments according to sub-region between control and OA individuals. Previous studies in cartilage have indicated that T1rho value changes may correlate with proteoglycan loss [8, 25] and changes in collagen architecture in vitro [26]. Our results suggest that in OA subjects, alterations in ligament composition occur heterogeneously within the ligament.

Previous histological studies have looked at composition of the ligaments and there has been interest in the biological mechanisms that underlie ligament degeneration in the cruciate ligaments. Hasegawa et al. found that disorganisation of collagen fibres was the first and most prevalent change in age-related degeneration of the anterior cruciate ligament [4]. Recent interest has been directed to how these compositional changes may differ through various sub-regions of the ligament. For example, a study by Skelley et al. documented how the anteromedial region of the ACL may have a different cellular composition to the posterolateral region [27]. A previous study by Wilson et al. investigated T2 mapping values in an asymptomatic sample and found significant differences between sub-regions of the PCL [14]. Namely, the distal third of the PCL had higher T2 values than the proximal or middle thirds. The present study similarly found variation between sub-regions, but in contrast found that the distal third of the PCL had lower T2 values than the proximal or middle. Despite this, the magnitude of the values was generally similar. This could be due to a number of reasons such as differences in the demographics of the cohort studied (the mean age in Wilson et al’s study was considerably younger at 39.8 years compared to 56 years in our healthy control group) or differences in the methodology of dividing the PCL into sub-regions; Wilson et al. used a 3D centreline. Another study found higher mean T2 values in the PCL than the mean values found in this study [15]. This could be explained by the present study investigating PCLs in asymptomatic individuals and those with OA, rather than those with PCL tears. Looking at the ACL, the magnitude of T2 relaxation values of healthy controls found in a previous study were generally similar to those found in the present study [16]. Quantitative MRI techniques such as those featured in this manuscript may allow for the further exploration of these sub-regional differences non-invasively.

### Future applications in in-vivo studies of the cruciate ligaments

Evaluating ligament healing in animal models currently requires histological or biochemical testing. These either require the need for a biopsy or destructive testing of the ligament [28]. Thus, in vivo assessment is impossible with these current methods. Previous studies looking at other MRI parameters, signal intensity or “grayscale” and volume of tissue in T2*-weighted MRI scans can predict structural properties of the healing ligament [29]. Another MRI variable, the T2* relaxation time variable, has been found previously to be linked to ligament structural properties; tissues with shorter T2* values were found to have higher ligament structural properties [30]. Further, shorter ligament T2* values are linked with higher histological scores of healing ligaments and could provide a method to assess ligament healing at a microscopic level [28]. These studies, together with our study working with T1rho and T2 parameters, reinforces the growing body of evidence that quantitative MRI methods could evaluate ligaments non-invasively in vivo.

### Future directions

The findings of this study are encouraging and should be replicated on a larger scale. A longitudinal study using a similar methodology to this study would help establish whether these T1rho and T2 mapping values could predict clinical outcomes over time and would provide more evidence that these non-invasive techniques can be used to track ligament degeneration or healing longitudinally. Further, studies monitoring repair tissue post ACL or PCL injury using these techniques could be possible.

### Limitations of this study

There are some important limitations in the present study that must be highlighted. First, this was a feasibility study and thus small in sample size, which creates an unavoidable consequence of an increased risk of a type II error. The relatively low number of control subjects (CON) compared to the osteoarthritis (OA) group in the study is also a limitation. However in the initial design phase of the study, it was felt the CON group was likely to be more homogenous than the OA and therefore a lower number of participants would be required, bearing in mind also the pragmatic considerations of availability of suitable age-matched healthy volunteers. This study’s findings also lack a histological correlation, which could validate how techniques are useful on a microscopic level. This would be difficult to obtain in humans in-vivo for practical and ethical reasons, however, a comparative assessment of histological and quantitative MRI features has previously been performed in-situ of cartilage and menisci in structurally intact human knee specimens [31]. Moreover, the methodology used for identification of the ACL and PCL used manual rather than automatic segmentation, allowing scope for human error. This being said, there is no established method of automatic segmentation and the fitting routine we used minimised error by ensuring exclusion of any pixels from the final map that could have been erroneous. Finally, the results of this study could have been influenced by the magic angle effect, whereby ordered collagen-containing tissues when oriented at 55 degrees to the direction of the magnetic field of the MRI scanner produce an artefactually increased signal [32]. Unfortunately, this may be difficult to avoid particularly in the PCL, given its natural curvature. Additionally, sequences were optimised for cartilage rather than ligament assessment. Further customisation (for example, of echo times) for ligament assessment may provide an advantage.

## Conclusion

This study shows that T1rho and T2 mapping of the cruciate ligaments is both feasible and reliable in subjects with OA and age-matched controls. We found significant variation between sub-regions of the cruciate ligaments in T1rho and T2 relaxation times for both controls and OA subjects, indicating heterogeneity across the ligaments. Significant differences between controls and OA subjects in this variation across the ligament was found. This study is an important step forward in identifying a non-invasive method to evaluate the cruciate ligaments in diseased human populations in vivo.

## Supplementary Information

**Additional file 1 **Comparing T1rho and T2 variables between OA individuals with a cyst and without. We compared the six individuals with OA who had a bone marrow lesion associated with the ACL/PCL tibial insertion to the nine other individuals with OA but no cyst. A Kruskal-Wallis test was used to compare the T1rho and T2 variables between these two groups. Supplementary Table 1 shows the mean ranks of each group for each variable. A significant difference between groups was found for the median T2 variable of the proximal part of the PCL (*p=*0.018). Comparing T1rho and T2 variables between OA individuals with KL Grade 3 and KL Grade 2. We compared the four individuals with a KL grade of 3 to the 11 with a grade of 2. Supplementary Table 2 shows the mean ranks of each group for each variable. A significant difference between groups was found for the median T2 variable of the middle part of the ACL (*p=*0.026). Overall, this study found a significant difference in one median T2 variable (the middle sub-region of the ACL) between individuals with a K-L grade 3 and those with a grade 2. Other comparisons in variables may have been limited by a lack of power, due to a very small sample size (there were only four individuals with a grade 3 knee). Most generally, we found that individuals with grade 3 knees had higher T1rho and T2 values than those with grade 2. We found a similar trend when comparing individuals with a cyst and without. Higher T1rho and T2 values have been previously associated with degenerative changes [33–35]. Thus collectively, more work is needed to clarify whether KL-grade and cyst presence is associated with these quantitative MRI values **Supplementary Table 1**: Summary of Kruskal-Wallis test of T1rho and T2 variables by sub-region between groups (OA with cyst vs OA without cyst)*.* **Supplementary Table 2**: Summary of Kruskal-Wallis test of T1rho and T2 variables by sub-region between groups (KL Grade 3 *vs* KL Grade 2).

## Data Availability

The datasets generated and analysed during the current study are not publicly available due to unattained permission from participants and research ethics committee but could be made available from JWM (email: jw.mackay@gmail.com).

## Abbreviations

ACL: Anterior Cruciate Ligament
ANOVA: Analysis of Variance
BMI: Body Mass Index
ICC: Intraclass correlation coefficient
KL: Kellgren Lawrence
KOOS-5: Knee Injury and Osteoarthritis Outcome Score
MRI: Magnetic Resonance Imaging
OA: Osteoarthritis
PCL: Posterior Cruciate Ligament
