# Supplementary material for: Quantitative analysis of the ACL and PCL using T1rho and T2 relaxation time mapping: an exploratory, cross-sectional comparison between OA and healthy control knees
Source: BMC Musculoskelet Disord. 2021 Oct 30;22:916. doi: 10.1186/s12891-021-04755-y (PMC8556921; doi:10.1186/s12891-021-04755-y)
Supplement: Supplementary file 1 — Additional file 1 Comparing T1rho and T2 variables between OA individuals with a cyst and without. We compared the six individuals with OA who had a bone marrow lesion associated with the ACL/PCL tibial insertion to the nine other individuals with OA but no cyst. A Kruskal-Wallis test was used to compare the T1rho and T2 variables between these two groups. Supplementary Table 1 shows the mean ranks of each group for each variable. A significant difference between groups was found for the median T2 variable of the proximal part of the PCL (p=0.018). Comparing T1rho and T2 variables between OA individuals with KL Grade 3 and KL Grade 2. We compared the four individuals with a KL grade of 3 to the 11 with a grade of 2. Supplementary Table 2 shows the mean ranks of each group for each variable. A significant difference between groups was found for the median T2 variable of the middle part of the ACL (p=0.026). Overall, this study found a significant difference in one median T2 variable (the middle sub-region of the ACL) between individuals with a K-L grade 3 and those with a grade 2. Other comparisons in variables may have been limited by a lack of power, due to a very small sample size (there were only four individuals with a grade 3 knee). Most generally, we found that individuals with grade 3 knees had higher T1rho and T2 values than those with grade 2. We found a similar trend when comparing individuals with a cyst and without. Higher T1rho and T2 values have been previously associated with degenerative changes [33–35]. Thus collectively, more work is needed to clarify whether KL-grade and cyst presence is associated with these quantitative MRI values Supplementary Table 1: Summary of Kruskal-Wallis test of T1rho and T2 variables by sub-region between groups (OA with cyst vs OA without cyst). Supplementary Table 2: Summary of Kruskal-Wallis test of T1rho and T2 variables by sub-region between groups (KL Grade 3 vs KL Grade 2). [file 12891_2021_4755_MOESM1_ESM.docx]

**Additional File 1**

**Comparing T1rho and T2 variables between OA individuals with a cyst and without**

We compared the six individuals with OA who had a bone marrow lesion associated with the ACL/PCL tibial insertion to the nine other individuals with OA but no cyst. A Kruskal-Wallis test was used to compare the T1rho and T2 variables between these two groups. Supplementary Table 1 shows the mean ranks of each group for each variable. A significant difference between groups was found for the median T2 variable of the proximal part of the PCL (*p=*0.018).

**Comparing T1rho and T2 variables between OA individuals with KL Grade 3 and KL Grade 2**

We compared the four individuals with a KL grade of 3 to the 11 with a grade of 2. Supplementary Table 2 shows the mean ranks of each group for each variable. A significant difference between groups was found for the median T2 variable of the middle part of the ACL (*p=*0.026).

Overall, this study found a significant difference in one median T2 variable (the middle sub-region of the ACL) between individuals with a K-L grade 3 and those with a grade 2. Other comparisons in variables may have been limited by a lack of power, due to a very small sample size (there were only four individuals with a grade 3 knee). Most generally, we found that individuals with grade 3 knees had higher T1rho and T2 values than those with grade 2. We found a similar trend when comparing individuals with a cyst and without. Higher T1rho and T2 values have been previously associated with degenerative changes (35), (36) and (37). Thus collectively, more work is needed to clarify whether KL-grade and cyst presence is associated with these quantitative MRI values.

**Supplementary Table 1:** Summary of Kruskal-Wallis test of T1rho and T2 variables by sub-region between groups (OA with cyst vs OA without cyst)

| Ligament | Parameter | Subregion | With cyst mean rank | Without cyst mean rank | Kruskal-Wallis H (df=1) | Sig. (two-tailed) |
| --- | --- | --- | --- | --- | --- | --- |
| ACL | T1rho | Distal | 10.0 | 6.67 | 2.00 | 0.157 |
|  |  | Middle | 10.0 | 6.67 | 2.00 | 0.157 |
|  |  | Proximal | 8.50 | 7.67 | 0.125 | 0.724 |
|  | T2 | Distal | 10.0 | 6.67 | 2.00 | 0.157 |
|  |  | Middle | 10.2 | 6.56 | 2.35 | 0.126 |
|  |  | Proximal | 8.33 | 7.78 | 0.0560 | 0.814 |
| PCL | T1rho | Distal | 7.83 | 8.11 | 0.0140 | 0.906 |
|  |  | Middle | 7.33 | 8.44 | 0.222 | 0.637 |
|  |  | Proximal | 9.67 | 6.89 | 1.39 | 0.239 |
|  | T2 | Distal | 8.00 | 8.00 | 0.00 | 1.00 |
|  |  | Middle | 10.3 | 6.44 | 2.72 | 0.0990 |
|  |  | Proximal | 11.3 | 5.78 | 5.56 | **0.0180** |

*NOTE: p-values < 0.05 are shown in* ***bold****.*

*With cyst = Individuals in the OA group with a cyst. Without cyst = Individuals in the OA group without a cyst. ACL = Anterior cruciate ligament; PCL = Posterior cruciate ligament.*

**Supplementary Table 2:** Summary of Kruskal-Wallis test of T1rho and T2 variables by sub-region between groups (KL Grade 3 vs KL Grade 2)

| Ligament | Parameter | Subregion | Grade 3 mean rank | Grade 2 mean rank | Kruskal-Wallis H (df=1) | Sig. (two-tailed) |
| --- | --- | --- | --- | --- | --- | --- |
| ACL | T1rho | Distal | 10.3 | 7.18 | 1.38 | 0.240 |
|  |  | Middle | 11.3 | 6.82 | 2.88 | 0.090 |
|  |  | Proximal | 9.00 | 7.64 | 0.273 | 0.602 |
|  | T2 | Distal | 10.3 | 7.28 | 1.38 | 0.240 |
|  |  | Middle | 12.3 | 6.45 | 4.93 | **0.026** |
|  |  | Proximal | 8.50 | 7.82 | 0.068 | 0.794 |
| PCL | T1rho | Distal | 10.5 | 7.09 | 1.71 | 0.192 |
|  |  | Middle | 7.50 | 8.18 | 0.068 | 0.794 |
|  |  | Proximal | 9.25 | 7.55 | 0.426 | 0.514 |
|  | T2 | Distal | 10.5 | 7.09 | 1.71 | 0.192 |
|  |  | Middle | 10.0 | 7.27 | 1.09 | 0.296 |
|  |  | Proximal | 10.8 | 7.00 | 2.06 | 0.151 |

*NOTE: p-values < 0.05 are shown in* ***bold****.*

*ACL = Anterior cruciate ligament; PCL = Posterior cruciate ligament.*
